# Extraordinary water adsorption characteristics of graphene oxide†
†Electronic supplementary information (ESI) available: Materials and Methods. Fig. S1 to S7. Table S1. See DOI: 10.1039/c8sc00545a


**DOI:** 10.1039/c8sc00545a

**Published:** 2018-05-16

**Authors:** B. Lian, S. De Luca, Y. You, S. Alwarappan, M. Yoshimura, V. Sahajwalla, S. C. Smith, G. Leslie, R. K. Joshi

**Affiliations:** a School of Chemical Engineering , University of New South Wales Sydney , Australia; b School of Materials Science and Engineering , University of New South Wales , Sydney , Australia . Email: r.joshi@unsw.edu.au; c CSIR – Central Electrochemical Research Institute , Karaikudi 630003 , Tamilnadu , India; d Surface Science Laboratory , Toyota Technological Institute , Nagoya , Japan

## Abstract

The laminated structure of graphene oxide (GO) confers unique interactions with water molecules which may be utilised in a range of applications that require materials with tuneable hygroscopic properties. The precise role of the expandable interlayer spacing and functional groups in GO laminates has not completely been understood to date. Herein, we report the experimental and theoretical investigations on the adsorption and desorption behaviour of water in GO laminates as a function of relative pressure. We observed that GO imparts high water uptake capacity of up to 0.58 gram of water per gram of GO (g g^–1^), which is significantly higher than silica gel as a conventional desiccant material. More interestingly, the adsorption and desorption kinetics of GO is five times higher than silica gel. The observed extraordinary adsorption/desorption rate can be attributed to the high capillary pressure in GO laminates as well as micro meter sized tunnel-like wrinkles located at the surface*.*

## Abstract 2

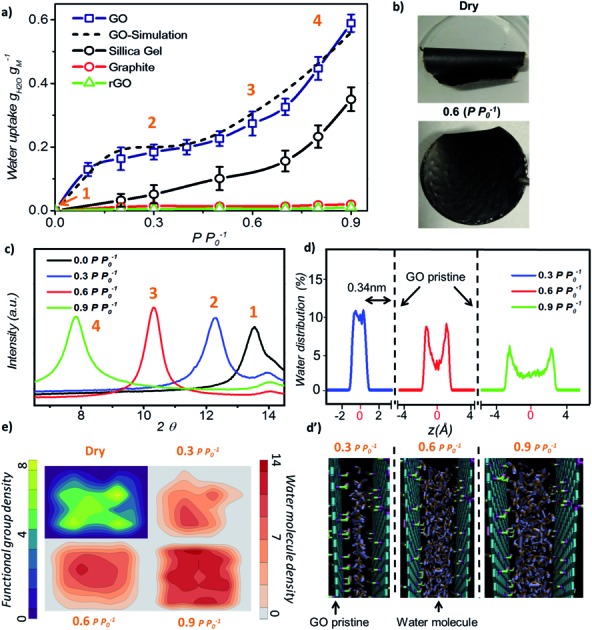
The laminated structure of graphene oxide (GO) confers unique interactions with water molecules which may be utilised in a range of applications that require materials with tuneable hygroscopic properties.

## 

Nanoporous materials with a high surface area and large pore volume are often employed as desiccant materials.^1^–^3^ Heterogeneous 3-D porous materials such as silica gel and zeolites are the widely used desiccant materials.^4^,^5^ However, issues such as large pore size distribution, low surface area to pore volume ratios, low hydrophilicity and/or poor hydrothermal stability associated with the aforementioned materials offer limitations for wide applicability.^6^–^8^ Although more recent advanced desiccant materials, such as MIL-type metal–organic frameworks (MOFs), have shown significant advancement in water adsorption capacities, yet their financial viability is relatively low for large productions.^9^,^10^ Recent studies on the interaction of water with graphene oxide laminates have demonstrated the possibility of utilizing their relatively high hydrophilicity for numerous applications.^11^–^13^ Being a 2-D porous material, graphene oxide not only possesses a more uniform pore size distribution but also has diverse functionalization potential, ultra-fast water transport mechanism and expandable interlayer spacing.^14^–^18^ All these features and the formation of a hydrogen bond network with water micro-clusters in the confined GO laminates significantly affects the diffusion rate of water molecules and potential energy at the absorbed state.^19^–^21^ The strong interaction between GO and water makes it a potential candidate for desiccant application. Herein, we studied the water adsorption capacity and kinetics of GO extensively.

Initially, we investigated the water vapour uptake of GO, silica gel, graphite and reduced graphene oxide (rGO) at different relative pressures *P P*_0_^–1^ (where *P*_0_ represents the saturation pressure) from 0.1 to 0.9. The results are summarised in Fig. 1. Both graphite and rGO show insignificant adsorption ability with values of less than 0.05 g g^–1^ water uptake (Fig. 1a). Although, the interlayer spacing of graphite (3.4 Å) and rGO (3.7 Å)^22^ should be enough to accommodate a 2.4 Å sized water molecule, the space is filled with electron density. Therefore, there exists no pore to accommodate the water molecule. Moreover, their hydrophobic characteristics also restrict the entry of water molecules into the pores of these materials.^23^ On the other hand, due to the larger proportion of hydrophilic functional groups, membrane-like GO prepared by vacuum filtration exhibits high adsorption capacity, which is at least two times higher than that of a conventional desiccant material such as silica gel (pore size 2–6 nm) across the tested range of relative pressure. The water uptake of GO is 0.13 g g^–1^ at a low relative pressure (*P P*_0_^–1^) of 0.1 and 25 *°*C, which reaches 0.58 g g^–1^ at a relative pressure of 0.9.

**Fig. 1:**
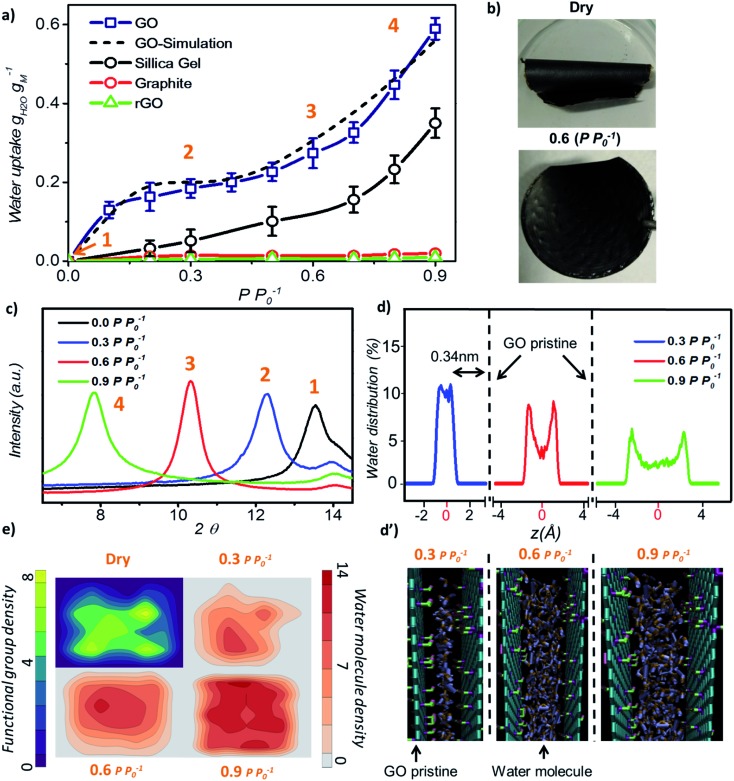
Water adsorption of graphene oxide. (a) Adsorption isotherms of GO, silica gel, graphite and rGO at 25 °C. (b) Photograph of GO laminates (top-dried at 80 °C and bottom-saturated at *P P*_0_^–1^ = 0.6). (c) XRD patterns of GO laminates under different conditions. (d) MD simulated water molecule distribution across the GO laminate at different relative pressures where *z* = 0 represents the centre of two GO planes and d′ shows the water molecule position across the two GO planes. (e) Distribution profile of water molecules and GO functional groups parallel to the GO plane.

In our study, the trend of the adsorption isotherm is in excellent agreement with the type-II isotherm classified by the International Union of Pure and Applied Chemistry (IUPAC).^24^ However, type II is often used to describe the adsorption behaviour of non-porous or macro-porous hydrophobic materials, while type I and IV are for microporous and mesoporous materials, respectively. This is contradictory to the existence of GO microporous structure and its hydrophilic nature. On the other hand, the traditional porous materials characterized by the IUPAC type have a rigid porous structure, while the interlayer spacing of GO can be varied under wet conditions. Fig. 1b shows the optical image of GO laminates in the membrane form at different hydration states. GO membranes dried at 80 °C for 10 min are folded whereas the wet membranes at a relative pressure of *P P*_0_^–1^ = 0.6 remain flat. Herein, the folding of GO membrane can be attributed to the severe contraction in the interlayer spacing of GO laminates at the membrane edge, thereby causing stress to appear on the sample surface. This was further confirmed from the X-ray diffraction (XRD) measurements as the *d*-spacing of GO sample changed from 6.5 Å under dry conditions to 11.3 Å at 0.9 *P P*_0_^–1^ (Fig. 1c). At relative pressures below 0.3, the *d*-spacing of GO was only enhanced by 1.8 Å as shown in the XRD plots, which is less than the size of a water molecule (∼2.4 Å). This suggests that the adsorption is mainly due to the strong interaction between water–GO surfaces that corresponds to the type I IUPAC adsorption isotherm reported for microporous materials. However, when the relative pressure increases above 0.6, the *d*-spacing was enhanced from 2.2 Å at *P P*_0_^–1^ = 0.6 to 4.8 Å at *P P*_0_^–1^ = 0.9, respectively, resulting in a proportional increase in the water uptake from 0.28 g g^–1^ to 0.58 g g^–1^. This evidenced the multilayer water formation in GO laminates by capillary condensation often exhibited by mesoporous materials in accordance with the IUPAC type IV adsorption isotherm (Fig. 1a). Thus, we believe that the adsorption isotherm of GO observed in this work is a combination of the IUPAC type I and type IV.

Further, we performed molecular dynamics (MD) simulations to probe more insights into the water behaviour within GO laminates under different relative pressure conditions. MD simulation accurately predicted the water adsorption capacity of GO with less than 7% deviation from the experimental results (Fig. 1a). The water molecule distribution profile in GO laminates obtained from the MD simulation was then plotted both along the thickness direction *z* (Fig. 1d and d′) and parallel to the GO basal plane (Fig. 1e). As depicted in Fig. 1d, over 90% of water molecules were positioned at ∼0.34 nm away from the pristine graphene plane at *P P*_0_^–1^ = 0.3.

Such a behaviour is due to the van der Waals force existing between water and pristine carbon to maintain low potential energy, and it shows the significance of surface water interaction at low relative pressure as demonstrated by the adsorption isotherm. The water distribution profile along the GO plane suggests that the water molecules were closely packed around the GO functional group at *P P*_0_^–1^ = 0*.*3 (Fig. 1e, top right) whereas a bimodal water molecule distribution was found for higher relative pressure (Fig. 1e, bottom). The average distance between the water molecule and GO functional group was calculated as 0.32 nm at 0.3 *P P*_0_^–1^ and 0.47 nm at 0.9 *P P*_0_^–1^. At *P P*_0_^–1^ = 0.9, a distinct bimodal distribution was noticed for water with two peaks positioned 0.34 nm apart from the GO basal plane, indicating the strong interaction between GO and water at high relative pressure. However, about 43% of water molecules are positioned away from the low potential energy free spots where water–water interaction dominates. This proves the capillary condensation effect inside GO laminates at high relative pressure.

During such circumstances, the free space between the individual GO sheets determines the adsorption capacity. This is also proved by the additional MD simulations; where more defects or fewer functional groups will increase the adsorption capacity of GO (Fig. S2†). In between these two stages, as the relative pressure increases from 0.3 to 0.6, water molecules start to occupy and as a result the GO capillary expands. The slower increase rate in the water uptake at this stage is due to the high potential mean force between GO laminates that quickly attains equilibrium with environmental water vapour pressure.^25^,^26^

In order to understand the kinetics of GO water adsorption and desorption, we measured the weight change of GO at 25 °C (ambient conditions) for the adsorption rate, and at 40 °C for the desorption rate (Fig. 2a). The overall adsorption rate of GO was calculated using the linear driving force model (LDF),^27^ where the rate of water adsorption is defined as 
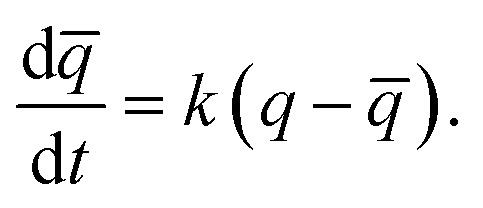
 Here *q[combining macron]* and *q* are the transient and equilibrium water adsorption in the sorbent, respectively, and *k* is the mass transfer coefficient. The average *k* value for silica gel was found to be 2.54 h^–1^, which in good agreement with the literature^28^ and similar to the *k* value measured on molecular sieves (3 Å).^29^ GO was found to have a *k* value of 13.36 h^–1^, which is around five times higher than that of silica gel, and is higher than that of MIL-101 without any modification by hydroscopic salts.^30^ We also studied the water adsorption capacity of grinded GO (average particle size ∼0.5 mm) and observed a higher adsorption rate which can be attributed to the more exposed GO surface area. However, the grinding process affects the integrity of the GO capillaries which leads to a slight decrease in the adsorption capacity by 0.01 g g^–1^.

**Fig. 2:**
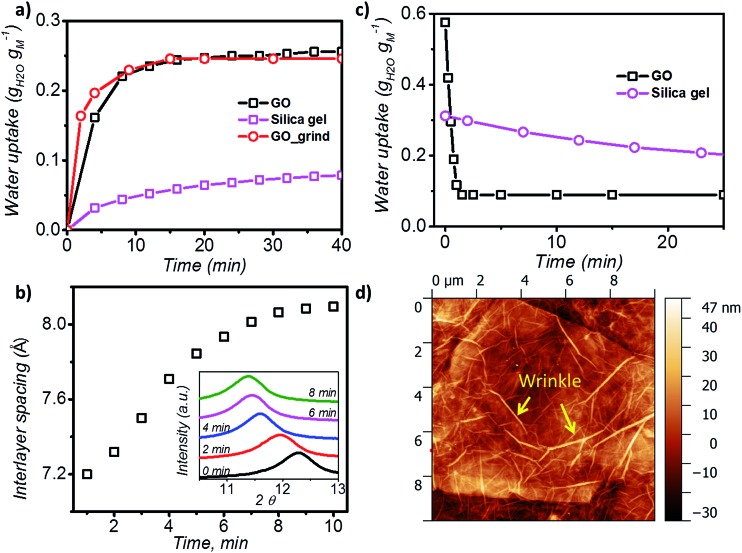
Adsorption/desorption kinetics of GO. (a) Water adsorption rate measured at 0.6 *P P*_0_^–1^ and 25 °C for 50 minutes. (b) Variation of interlayer spacing of GO laminates with time; the inset shows the XRD plots during the adsorption process of GO (in the range 2*θ* = 6° to 16°). (c) Water desorption rate at 40 °C and 0.2 *P P*_0_^–1^ (the GO sample was saturated at *P P*_0_^–1^ = 0.9 overnight prior to the water desorption). (d) AFM images of GO showing 20 nm tunnel-like wrinkles.

XRD was further utilised to analyse the expansion of *d*-spacing during GO water adsorption. In our XRD measurements, 10 individual scans for 2*θ* = 6° to 16° were performed continuously for 10 minutes during the water adsorption of GO by varying the relative pressure from 0.3 to 0.53 (Fig. 2b). We observed expansion of *d*-spacing from 7.2 Å to 7.9 Å with a constant increase rate of 0.1 Å min^–1^ in the first 7 minutes of water adsorption, before reaching the equilibrium *d* value of 8.1 Å for GO. It is also interesting to note that during the water adsorption process, XRD always shows one distinct peak shift rather than the exchange of two peaks. This confirms that the expansion of GO laminates is a collective movement of all laminates across the entire GO membrane.

During desorption experiments, we noticed that about 80% of water (by weight) desorbed at a low regeneration temperature of 40 °C with a very fast desorption rate of 0.46 g g^–1^ per minute (Fig. 2c). The fast desorption rate of GO is due to the combined effect of relatively higher temperature and low humidity. The moderate temperature of 40 °C can accelerate the diffusion rate of water molecules and the low relative pressure creates a larger pressure difference to further push water out of the GO capillaries. GO also showed a consistent lower water uptake than silica gel at tested regeneration temperatures (40 to 100 °C) (Fig. S3†), and exhibited excellent stability (Fig. S4†), thereby proving it as an ideal material for the desiccation process.

To understand the rapid water adsorption and desorption rate of GO, MD simulation for GO water adsorption was performed. The simulation results for water adsorption kinetics confirmed that all water molecules are absorbed into the capillaries inside the GO laminates within the first 1.5 ns and no water molecule comes out for 20 ns (Fig. S5†). This indicates the existence of a very strong capillary pressure in the GO even at low water uptake. It also shows that the rate of water adsorption is not only limited by the speed of water entering into open GO pores but also controlled by the rate of water transport inside GO laminates. A common term that represents the molecular mobility in the nanoporous structure in MD simulations is the self-diffusivity *D*. The simulated value of *D* for water molecules in GO at *P P*_0_^–1^ = 0.6 was equal to 0.131 × 10^–9^ m^2^ s^–1^ (Fig. S6†), which is in agreement with the values reported by Jiao *et al.* (∼0.147 × 10^–9^ m^2^ s^–1^) and Devanathan *et al.* (∼0.15 × 10^–9^ m^2^ s^–1^).^31^,^32^ In comparison with the reported diffusivity of water in silica gel (in the range 0.28 × 10^–9^ m^2^ s^–1^ to 1.5 × 10^–9^ m^2^ s^–1^)^33^ the simulated *D* value for water in GO laminates suggests low mobility based self-diffusion which is contrary to our experimental results. Further experiments are mandatory to describe the fast water transport in GO.

Analysis of the surface morphology using atomic force microscopy (AFM) further helps to understand the rapid water adsorption and desorption of GO. We observed wrinkle (micrometre size)-like structures with an average height of 20 nm and up to 1.5 μm in size on the GO surface (Fig. 2d and S7†). Such winkles were also observed in the literature for thin graphene and GO membranes.^34^,^35^ Wang *et al.* further confirmed that the formation of the wrinkle-like structure on graphene oxide is caused by drying, and the structure is not a temporary elastic deformation but is permanent.^36^ These wrinkle-like tunnels were nanosized airbags trapped within the GO membrane due to the stacking of graphene oxide laminates during the vacuum filtration process. The GO membranes were made by a vacuum filtration method which relies on the applied vacuum force to drag each GO monoflake down to the target substrate and assemble layer by layer to form the thin layer. However, in the GO solution, the GO nanoflakes were not completely flat but in a corrugated form. Also, the applied vacuum pressure cannot be uniformly distributed along the substrate. Therefore, nanosized wrinkles or folds will be easily formed along the GO flakes. As it will be laminated layer by layer, any small wrinkles or folds can be cumulated as a microsized wrinkle channel in the final GO membranes.

Such winkles may act as channels to rapidly distribute water through the GO structures, allowing shorter diffusion pathways through the gallery spaces between wrinkles. However, more detailed investigations about this structure are required, especially its distribution when embedded under the GO membrane surface.

Furthermore, the adsorption isotherm (Fig. 3) at 25 °C and 40 °C for the GO membrane was measured, and the adsorption enthalpy was calculated using the Clausius–Clapeyron relation.1
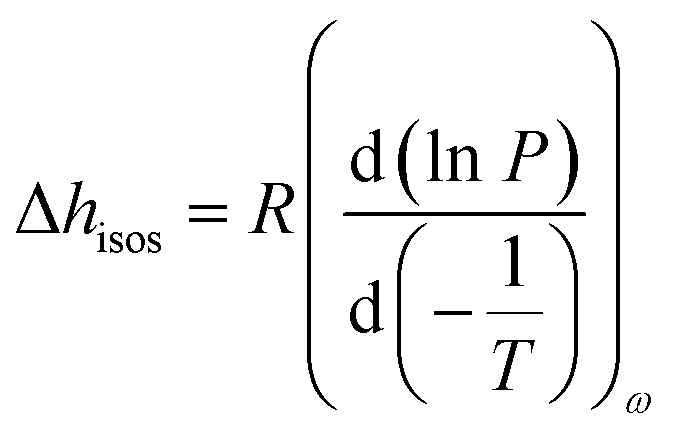
where Δ*h*_isos_, *R*, *P*, *T*, and *ω* represent the isosteric enthalpy of adsorption, universal gas constant, pressure, temperature, and water uptake, respectively. The isosteric enthalpy of water adsorption is 30% higher than that of silica gels,^37^ and is on the same level as desiccants with high adsorption enthalpies, such as zeolite 13X.^38^ The simulated enthalpy change during adsorption was equal to –4062.3 kJ kg^–1^, and it is in excellent agreement with the experimental data which further validates our MD module. Such a property is good for applications such as heat pump.

**Fig. 3:**
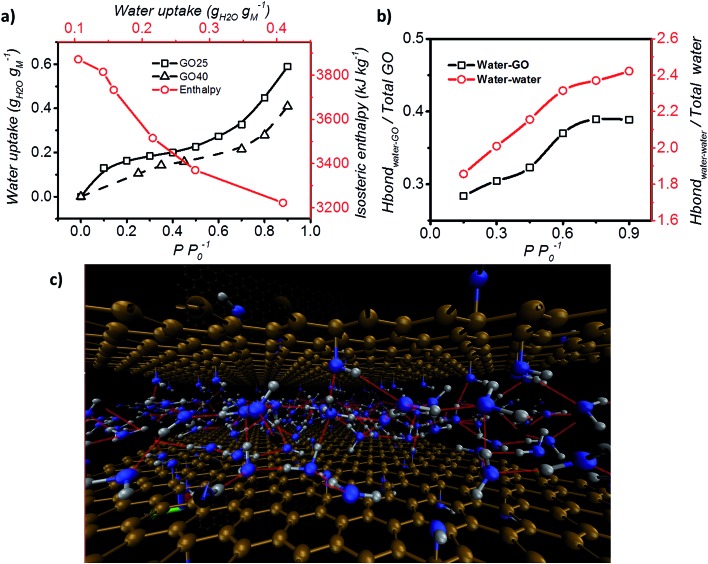
Adsorption enthalpy and hydrogen bonding network. (a) Adsorption isotherm of GO measured at both 25 °C and 40 °C, and the isosteric enthalpy of adsorption calculated based on the Clausius–Clapeyron relation. (b) Representation of the interaction between water–GO functional group (*Y* axis – left) and water–water (*Y* axis – right) at different relative pressures. (c) Schematic of the simulated GO–water configuration and hydrogen bond network at 0.6 *P P*_0_^–1^. C, O and H are shown in brown, blue and grey and hydrogen bonds are represented by red lines (picture obtained with Visual Molecular Dynamics, VMD^40^).

Hydrogen bond analysis based on MD simulation confirmed that the interaction amongst water molecules is stronger than the interaction between water molecules and GO at high relative partial pressure (Fig. 3b). The ratio of the functional groups that form hydrogen bonds with water was found to increase from 0.27 to 0.37 with increasing humidity (from 0.15 to 0.75 *P P*_0_^–1^). However, saturation occurs under conditions above *P P*_0_^–1^ = 0.75. Under such conditions, the number of functional groups that are expected to form hydrogen bonds with water remains fixed. In contrast, the hydrogen bond between water molecules continuously increases from 1.9 to 2.4 hydrogen bonds per water molecule. This is expected, as the increase in the humidity expands the *d* spacing of GO laminates and this enhances the number of water molecules exposed to each other leading to a higher number of hydrogen bonds per water molecule. However, after reaching a relative pressure (*P P*_0_^–1^) of 0.6, the hydrogen bonds saturate at a value of 2 to 3 hydrogen bonds per water. Below a relative pressure (*P P*_0_^–1^) of 0.3, the simulated average number (∼2) of hydrogen bonds is lower than the reported 2.3 hydrogen bonds per water molecule for liquid water.^39^ This indicates that water may exist in an intermediate state between liquid and vapour.

In conclusion, GO laminates demonstrate remarkable water adsorption characteristics. The high water uptake capacity of GO is due to its expandable 2D porous laminated structure, and the fast water adsorption/desorption ability of GO can be attributed to the existence of wrinkle-like water tunnels. Comparing this with the commonly used desiccant material (silica gel), GO has higher water uptake, a rapid adsorption/desorption rate and higher adsorption enthalpy. Its thin film form also facilitates its easy accessibility to a rotary desiccant device with a belt or wheel disk. These characteristics make it an advanced material for desiccant application as well as for heat pump processes.

A limitation which might be accompanied with the commercial use of GO as a desiccant material can be the cost of production in mass scale as compared to silica gel, however, the superior properties of GO make is useful for applications where efficacy is significantly important. GO membranes are in laminated form whereas the common desiccant material (such as silica gel) is usually made into a 3-dimensional pellet structure. We believe that dramatic progress can be made by producing GO with a 3-dimensional structure.

## Conflicts of interest

There are no conflicts to declare.

## Supplementary Material

Supplementary information
