# Supplementary material for: Extraordinary water adsorption characteristics of graphene oxide
Source: Chem Sci. 2018 May 16;9(22):5106–11. doi: 10.1039/c8sc00545a (PMC5994872; doi:10.1039/c8sc00545a)
Supplement: Supplementary file 1 [file SC-009-C8SC00545A-s001.pdf]

Electronic Supplementary Material (ESI) for Chemical Science.
This journal is © The Royal Society of Chemistry 2018

Supplementary Information (SI)

Extraordinary water adsorption characteristics of graphene oxide

B. Lian[1], S. De Luca[1], Y. You[2], S. Alwarappan[3], M. Yoshimura[4], V. Sahajwalla[2], S.C. Smith[1], G. Leslie[1], *R.K. Joshi[2]

[1]School of Chemical Engineering, University of New South Wales Sydney, Australia.
[2]School of Materials Science and Engineering, University of New South Wales, Sydney Australia
[3]CSIR- Central Electrochemical Research Institute, Karaikudi 630003, Tamilnadu, India.
[4]Surface Science Laboratory, Toyota Technological Institute, Nagoya, Japan.
[5]Department of Chemistry and School of Materials Science, Ulsan National Institute of Science and Technology, Ulsan, South Korea
***Corresponding author's email:* r.joshi@unsw.edu.au**

.

Methods:

GO membrane preparation:

Graphene oxide was prepared using Hummer's method [1]. Briefly, 1.0 g of sodium nitrite and 2.0 g of graphite flakes with the size of 0.5 µm (from Sigma-Aldrich) is stirred with 48 mL of Con. H_2SO_4 in an ice bath. 4.6 g of $KMnO_4$ was then slowly added to the suspension to maintain temperature of the mixture below 15°C. The mixture was then stirred at room temperature for 30 mins, before being diluted using 100 mL Milli-Q water. The reaction vessel was kept at 98°C for half an hour and then 100 mL of 2% hydrogen peroxide solution was added. The solid in the suspension then gets separated and washed using 1.5 L of Milli-Q water GO membrane were prepared by vacuum filtration of the resulting GO suspension through a 0.2 µm Polyvinylidene fluoride membrane. rGO was prepared by soaking GO membrane in 10% hydroiodic acid for 4 hours. The resulted sample showed bright reflection of light and a hydrophobic feature.

Adsorption of water vapour in GO:

Adsorption equilibrium experiments were performed at atmospheric pressure in an Environmental chamber. The relative pressure was controlled to ±3% and the temperature could be controlled to an accuracy of ±1 K. A microelectronic balance with an accuracy of 0.0001 g was used to measure the sample weight. The disk like GO membrane has a radius

of 4cm and a thickness of 4-7μm. the grinded GO with similar thickness but significant lower radius of around 3-5mm. The granular silica gel was purchased from Sigma-Aldrich with pore size of 2.4nm and particle size of 0.2mm to 1mm. 0.05 g dry mass of every prepared adsorbent was placed or spread evenly on a Petri-dish with 10cm in diameter. The samples were pre-heated for 10 minutes and 30 minutes for GO and silica gel respectively at 80°C to discharge excess water prior testing at different temperatures and humidity. To measure the adsorption isotherm, all samples were kept in the environment chamber for at least 40 minutes. The sample weight was constantly inspected until no more weight change was noticed to ensure that the sample reach the adsorption equilibrium. Due to the fast water adsorption rate, GO membrane is expected to be applied to a moderate to high humidity environment for fast adsorption/desorption cycle. Thus, water adsorption rate was measured by recording the weight change of samples at 25°C and moderate humidity of $P\ P_0^{-1} = 0.53$, after being dried at 80°C. The samples were then placed in a desiccator for cooling prior to the weight measurement to illuminate heat effect on mass balance.

Simulation methods:

Classical molecular dynamics (MD) simulations were performed using the large-scale atomic/molecular massively parallel simulator (LAMMPS) [2]. The all-atom optimized potentials for liquid simulations (OPLS-AA) [3] were used for GO, which can capture essential many-body terms in interatomic interactions, including bond stretching, bond angle bending, nonbonding van der Waals and electrostatic interactions [4]. Three planar GO sheet (5*5 nm) with functional groups randomly seeded on the plane was created using MOLTEMPLATE [5]. Four graphene walls were set at the edge of upper and lower GO sheet to ensure water absorbed only in the two GO laminates. SPC/E model [6] was selected for water molecule with SHAKE algorithm [7] for constraining bond and angle. The interaction between water molecules and graphene or GO sheets includes both van der Waals and electrostatic terms. The former choice is described by the 12−6 Lennard−Jones potential VLJ = 4ε[(σ/r)12 − (σ/r)6] as a function of the interatomic distance r, with interaction parameters between water molecules and carbon atoms and functional group of GO are ϵ = 0.09369 kcal mol^{-1}, σ = 3.19 Å and ϵ = 0.1553 kcal mol^{-1}, σ = 3.17 Å respectively [8]. The partial charge of hydrogen, carbon and oxygen from GO was set to be 0.3294e, 0.1966e and -0.526e respectively [9]. The van der Waals forces are truncated at a distance of 1nm with a

constant shift in the energy over the whole range to remove the discontinuity. The long-range Coulombic interactions were computed using the particle–particle particle-mesh algorithm (PPPM) [10].

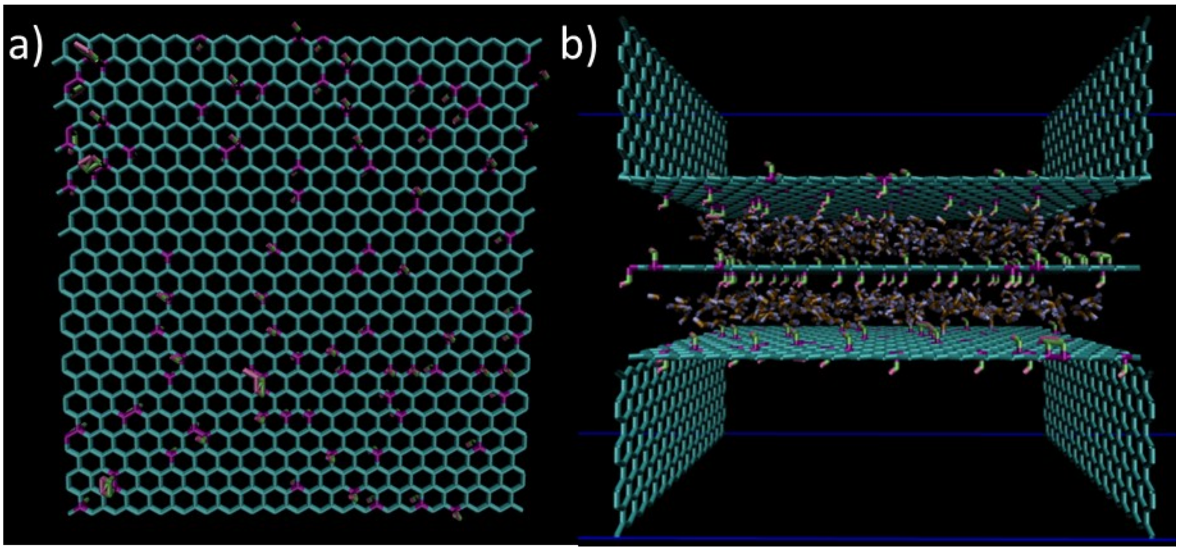

Figure s1: *MD simulation geometry with a) single GO flake at 10% oxidation level, functional group facing both side of the flake, b) 3 GO flake forming and water molecule in the two GO laminates. Four graphene walls were the constructed at the edge of the GO flake to prevent water molecule trapped at below and above the GO laminates in the simulation box.*

A simulation box of 5×5×4.5 nm was employed with periodic boundary condition applied at all directions (Figure s1). Detailed simulation geometry is shown in the supplementary information (Table s1). The simulation time step of 1 fs was selected for the integration of equation of motion. The overall system was minimized for 1000-time steps prior simulation and was electro-neutral to guarantee the convergence of the Ewald sum.

In order to predict the water uptake at different humid conditions, the simulations are carried out in the NVT ensemble with the Nosé–Hoover thermostat at 300K. Preselected interlayer spacing (based on XRD results) was used at different RH. The amount of water kept inside the GO laminate at the beginning of the simulation were continuously varied until no water molecule come out of the GO sheet until 10ns. 10% functional group as well as no defects was selected as default, while the variation of these two values was simulated under $P\,P_0^{-1} = 0.6$.

The self-diffusion coefficient of water was calculated from the trajectory of water atom inside GO laminates at equilibrium state by using Einstein's correlation function between atomic position and diffusivity D = limt →∞ (|r(t) – r(0)|)/2dit, where r is the position of the atom, t is simulation time step and di is the dimensions of space for water movement. Due to the 2D porous natural of GO, water movement in the normal direction is limited compare to the in-plane motion. So the di = 2 was considered in this simulation [11]. The diffusivity was calculated using results from 0.5 ns after the simulation system reach equilibrium to ensure an accurate estimation of the diffusion constant. The adsorption enthalpy was calculated by comparing the total energy of water at a constant relative pressure and at the equilibrium state (when absorbed in GO laminates).

Additional results

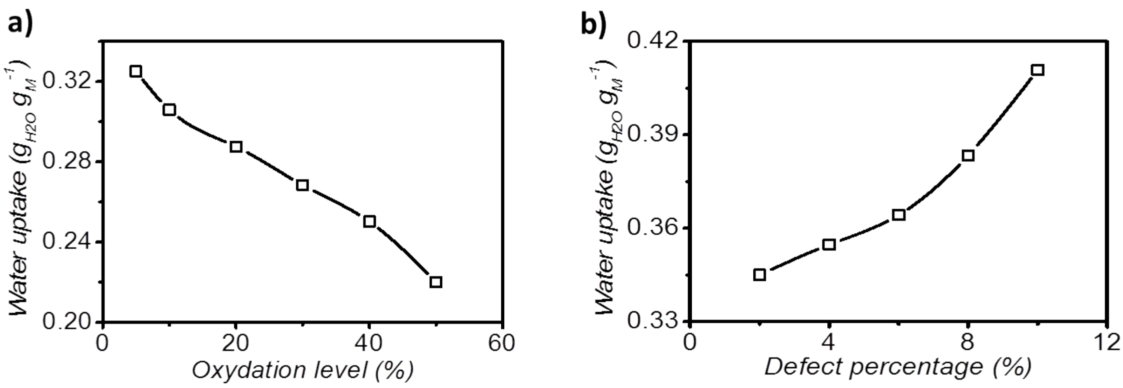

Figure s2: MD simulation predicted water uptake for modified GO membrane with a) different oxidation level, b) defect percentage

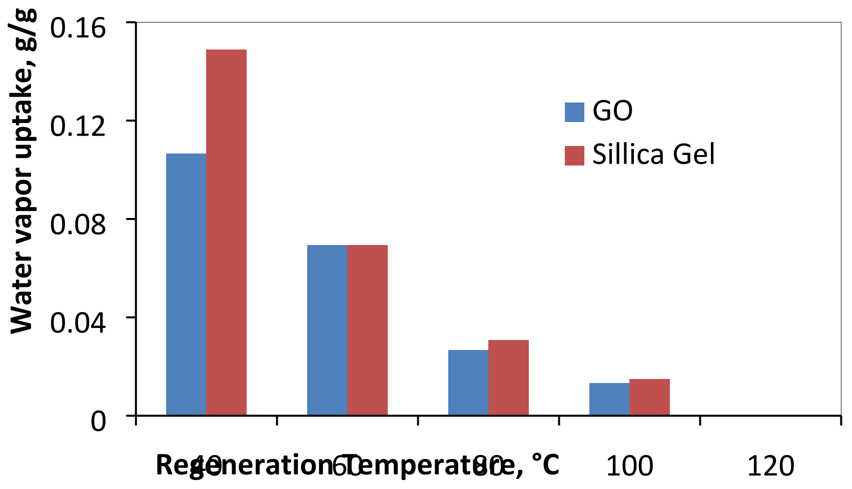

Figure s3: GO and silica gel's water uptake at different desorption temperatures

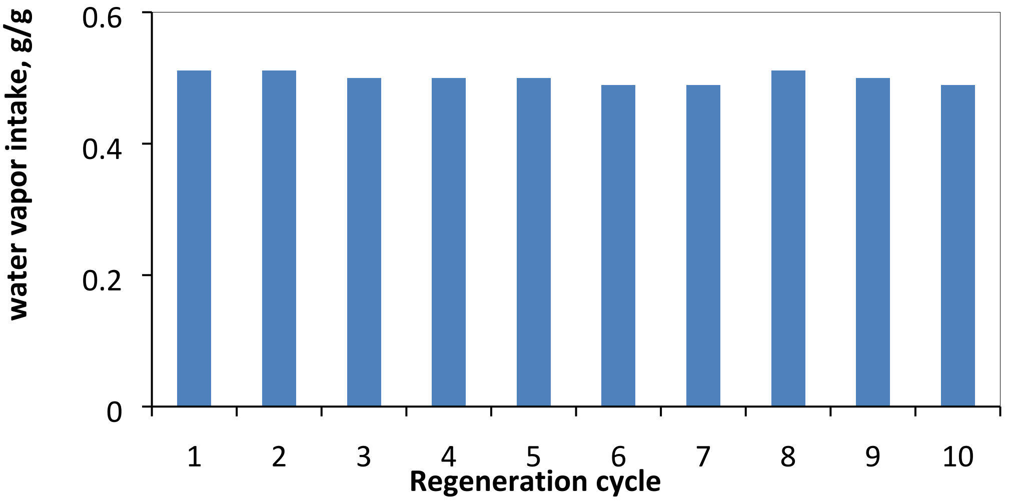

Figure s4: GO water adsorption capacity over 10 cycles of regeneration.

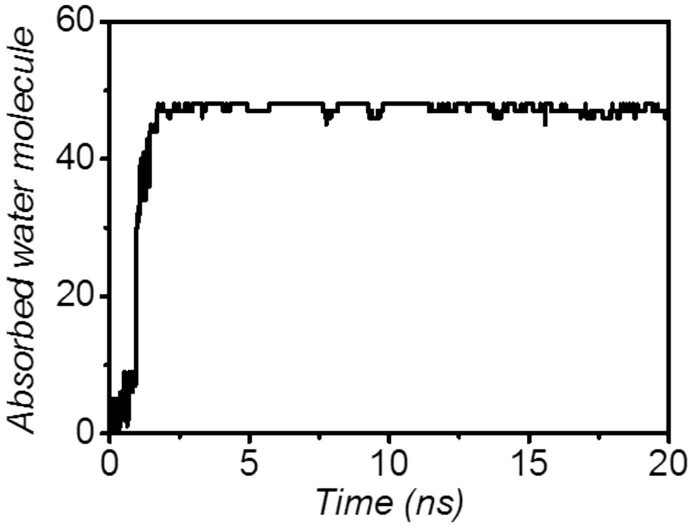

Figure s5: The number of water molecules (over 48 total water molecules) absorbed by GO as a function of time. The simulation was set at 60% relative humidity with a d-spacing of 8.6 Å for GO membrane.

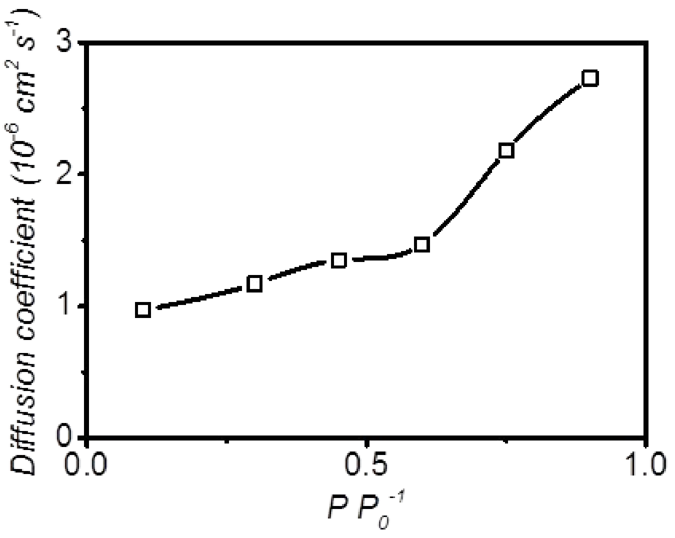

Figure s6: Simulated water molecule diffusion coefficient in GO at different humidity.

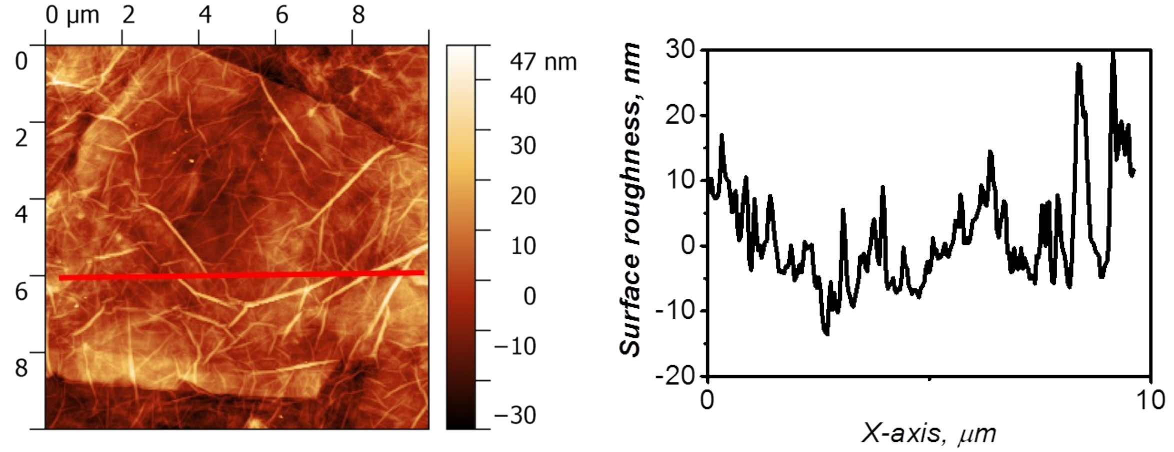

Figure s7: Surface roughness of GO at red line shown in AFM image (figure 2d main text)

Table s1: Summary of MD simulation for GO water adsorption at different relative pressure

Description	Value		
No. Flake	3		
GO flack dimension	5nm*5nm		
Total carbon in GO	2880		
Total functional group in GO	288		
Relative pressure, *P* P_0^{-1}	0.15	0.3	0.45
D spacing of GO, nm	0.69	0.76	0.8
No. Water molecule absorbed	390	507	567
P/P_0	0.6	0.75	0.9
D spacing of GO, nm	0.86	0.96	11.3
No. Water molecule absorbed	672	860	1268

References

[1] W. S. Hummers and R. E. Offeman, “Preparation of Graphitic Oxide,” *J. Am. Chem. Soc.*, vol. 80, no. 6, p. 1339, 1958.

[2] S. Plimpton, “Fast Parallel Algorithms for Short-Range Molecular Dynamics,” *J. Comput. Phys.*, vol. 117, no. 1, pp. 1–19, Mar. 1995.

[3] W. L. Jorgensen, D. S. Maxwell, and J. Tirado-Rives, “Development and Testing of the OPLS All-Atom Force Field on Conformational Energetics and Properties of Organic Liquids,” *J. Am. Chem. Soc.*, vol. 118, no. 45, pp. 11225–11236, 1996.

[4] C.-J. Shih, S. Lin, R. Sharma, M. S. Strano, and D. Blankschtein, “Understanding the pH-Dependent Behavior of Graphene Oxide Aqueous Solutions: A Comparative Experimental and Molecular Dynamics Simulation Study,” *Langmuir*, vol. 28, no. 1, pp. 235–241, 2012.

[5] A. Jewett, “A cross-platform text-based molecule builder,” *Moltemplate*, 2017. [Online]. Available: http://www.moltemplate.org/.

[6] H. J. C. Berendsen, J. R. Grigera, and T. P. Straatsma, “The missing term in effective pair potentials,” *J. Phys. Chem.*, vol. 91, no. 24, pp. 6269–6271, 1987.

[7] J.-P. Ryckaert, G. Ciccotti, and H. J. . Berendsen, “Numerical integration of the cartesian equations of motion of a system with constraints: molecular dynamics of n-alkanes,” *J. Comput. Phys.*, vol. 23, no. 3, pp. 327–341, Mar. 1977.

[8] T. Werder, J. H. Walther, R. L. Jaffe, T. Halicioglu, and P. Koumoutsakos, “On the Water−Carbon Interaction for Use in Molecular Dynamics Simulations of Graphite and Carbon Nanotubes,” *J. Phys. Chem. B*, vol. 107, no. 6, pp. 1345–1352, 2003.

[9] S. Jiao and Z. Xu, “Selective Gas Diffusion in Graphene Oxides Membranes: A Molecular Dynamics Simulations Study,” *ACS Appl. Mater. Interfaces*, vol. 7, no. 17, pp. 9052–9059, 2015.

[10] R. W. Hockney and J. W. Eastwood, *Computer Simulation Using Particle*. Taylor & Francis, 1989.

[11] J. Mittal, T. M. Truskett, J. R. Errington, and G. Hummer, “Layering and Position-Dependent Diffusive Dynamics of Confined Fluids,” *Phys. Rev. Lett.*, vol. 100, no. 14, p. 145901, Apr. 2008.
